# Neuropsychiatric disorders in Cushing's syndrome

**DOI:** 10.3389/fnins.2015.00129

**Published:** 2015-04-20

**Authors:** Rosario Pivonello, Chiara Simeoli, Maria Cristina De Martino, Alessia Cozzolino, Monica De Leo, Davide Iacuaniello, Claudia Pivonello, Mariarosaria Negri, Maria Teresa Pellecchia, Felice Iasevoli, Annamaria Colao

**Affiliations:** ^1^Dipartimento di Medicina Clinica e Chirurgia, Sezione di Endocrinologia, Università “Federico II”Naples, Italy; ^2^Department of Medicine and Surgery, Center for Neurodegenerative Diseases, University of SalernoSalerno, Italy; ^3^Laboratory of Molecular and Translational Psychiatry, Department of Neuroscience, Università “Federico II”Naples, Italy

**Keywords:** Cushing's syndrome, psychiatric disorders, neurological disorders, cognitive impairment, quality of life

## Abstract

Endogenous Cushing's syndrome (CS), a rare endocrine disorder characterized by cortisol hypersecretion, is associated with psychiatric and neurocognitive disorders. Major depression, mania, anxiety, and neurocognitive impairment are the most important clinical abnormalities. Moreover, patients most often complain of impairment in quality of life, interference with family life, social, and work performance. Surprisingly, after hypercortisolism resolution, despite the improvement of the overall prevalence of psychiatric and neurocognitive disorders, the brain volume loss at least partially persists and it should be noted that some patients may still display depression, anxiety, panic disorders, and neurocognitive impairment. This brief review aimed at describing the prevalence of psychiatric and neurocognitive disorders and their characterization both during the active and remission phases of CS. The last section of this review is dedicated to quality of life, impaired during active CS and only partially resolved after resolution of hypercortisolism.

## Introduction

Chronic glucocorticoid (GC) overproduction by the adrenal glands represents the cause of endogenous Cushing's syndrome (CS), a rare but serious endocrine disorder. In approximately 80% of cases, endogenous CS is consequence of an ACTH hypersecretion (ACTH-dependent CS), generally due to a corticotroph pituitary tumor (Pituitary-dependent CS, or Cushing's disease, CD), and rarely due to an ACTH-secreting or CRH-secreting extra-pituitary tumor (Ectopic Cushing Syndrome, ECS), whereas in the remaining 20% of cases CS is independent from ACTH hypersecretion (ACTH-independent CS) and GC excess is direct consequence of autonomous overproduction by the adrenal glands, because of unilateral adrenocortical tumors or bilateral adrenal hyperplasia, or dysplasia (Arnaldi et al., 2003; Newell-Price et al., 2006; Pivonello et al., 2008). Chronic endogenous GC exposure determines several clinical complications, including metabolic complications such as visceral obesity, insulin resistance with glucose intolerance and diabetes mellitus, and dyslipidemia, which configure a metabolic syndrome, cardiovascular complications such as systemic arterial hypertension, atherosclerosis, thromboembolism, bone complications such as osteoporosis and osteoarthritis, infective complications, ranging from an increase susceptibility to infections up to a fatal sepsis, as well as neuropsychiatric disorders (Pivonello et al., 2005, 2010; Tauchmanovà et al., 2006; De Leo et al., 2010; Pereira et al., 2010; van der Pas et al., 2013). These clinical complications negatively impact the quality of life (QoL) of patients with CS and increase their morbidity and mortality, mainly due to cardiovascular and infective diseases (Gotch, 1994; Webb et al., 2008; Clayton, 2010). The current management of patients with CS cannot ignore psychiatric and neurocognitive disorders, their evolution during the active disease and after disease remission, and their impact on QoL. The first description of neuropsychiatric disturbances in CS occurred in 1932 when, in his original description of a series of 12 CS cases, Harvey Cushing highlighted the presence of “emotional disturbances” as a pathologic feature of CS (Cushing, 1932). During the following years, several studies were performed with the purpose to better characterize the spectrum and the frequency of neuropsychiatric disorders in patients with CS (Sonino and Fava, 2001). These disorders include psychiatric disorders such as major depression, mania and anxiety, and neurocognitive disorders, mainly characterized by impairment of memory and concentration (Dorn et al., 1997; Sonino and Fava, 2001). Neuropsychiatric disorders contribute to significantly impair health-related QoL (Gotch, 1994; Webb et al., 2008). The aim of this brief review was to describe the main psychiatric and neurocognitive disorders observed in patients with endogenous CS during active disease and their development after disease remission, also discussing their impact on QoL. Additionally, this brief review aimed at addressing a simple overview on this topic, highlighting the important role of long-term follow-up and careful periodical investigation of psychiatric and neurocognitive disorders in the management of CS both in the active phase and after disease remission.

## Neuropsychiatric disorders during active disease

GCs have a crucial role in stress response and GC receptors have a pleiotropic distribution in central nervous system (CNS), mainly in the hippocampus; therefore, it is not surprising that chronic GC excess can lead to structural and functional changes in CNS, which are mainly based on brain atrophy (De Kloet et al., 1998; Sonino et al., 2010). The mechanisms by which GCs induce brain damage are largely unknown although four theories have been suggested. (1) The decrease of glucose uptake is responsible of brain atrophy. According with this theory, GCs induce brain damage by reducing glucose utilization in brain, as supported by the evidence of a generalized reduction in cerebral glucose metabolism in all areas of the brain in patients with CD. (2) The increase of excitatory aminoacids is responsible of toxic effects on nervous cells. GCs increase the release or enhance the effects of excitatory aminoacids, such as glutamate, which cause cell damage inducing the dendritic atrophy, particularly in the hippocampus. (3) The inhibition of “long-term potentiation” is responsible of cognitive deficits. GCs reduce the synthesis of neurotrophic factors, such as nerve growth factor-b and brain-derived neurotrophic factor, which through a presynaptic mechanism inhibit the long-term potentiation that is believed to be the mechanism behind learning process and memory formation. Additionally, this neurotrophic factors reduction can also be responsible of brain atrophy. (4) The suppression of neurogenesis in the dentate gyrus. GCs excess may suppress neurogenesis in the dentate gyrus, which determine hippocampal volume loss (De Kloet et al., 1998; Jacobs et al., 2000; Simmons et al., 2000; Bourdeau et al., 2002; Patil et al., 2007; Michaud et al., 2009). All these mechanisms seem to explain the GC-induced brain and, mainly, hippocampal damage responsible for neurocognitive disorders, although the atrophy of the prefrontal cortex (Patil et al., 2007) or the suppression of neurogenesis in the dentate gyrus (Jacobs et al., 2000) have been specifically identified as crucial pathophysiological events for the development of depression.

The most important psychiatric disorders observed in patients with active CS are: (1) major depression, which include alterations in mood, affective, vegetative, and cognitive functions; and (2) mania and anxiety. Major depression represents the most frequent and severe psychiatric disorder associated with chronic endogenous hypercortisolism. The prevalence of major depression in patients with CS is reported to be about 50–81%, as summarized in Table 1 (Haskett, 1985; Hudson et al., 1987; Loosen et al., 1992; Sonino et al., 1993, 1998; Dorn et al., 1995; Kelly, 1996). Focusing on CD, Sonino and co-workers reported major depression to occur in 54% of 162 patients and observed that it was significantly associated with female gender, older ages, higher urinary cortisol levels, relatively more severe clinical conditions and undetectable pituitary tumor (Sonino et al., 1998). Different degrees of severity were reported, ranging from latent to very severe melanchonic forms, also associated with suicide thoughts and attempts (Starkman et al., 1981; Starkman, 2013). Therefore, a careful periodic investigation of psychiatric symptoms should be performed in all patients with active CS, also taking in consideration that depression in these patients is typically characterized by intermittent phases, with episodes of exacerbation recurring very frequently at irregular intervals (Starkman et al., 1981; Starkman, 2013). Additionally, all domains of the depressive syndrome including mood, affect, vegetative, and cognitive functions can be compromised (Starkman, 2013). As far as mood alterations are concerned, many patients with CS up to 86%, show irritability, which often has early onset, appearing even before than other clinical symptoms and signs (Starkman et al., 1981; Starkman, 2013). It has been reported that patients with CS described themselves as having an increased sensitivity and they perceived themselves unable to ignore minor irritations and feeling “impatient,” with “over-reactivity,” generalized hypersensitivity to stimuli and an easy development of anger (Starkman et al., 1981). Depressed mood has been reported in 74% of CS patients, without regular cyclicity, although a common depressive episode persists usually 1–2 days and rarely more than 3 days per week (Starkman, 2013). In some patients depressed mood could be present on awakening and remain throughout the day or the next day as well (Starkman et al., 1981); in other patients, the onset of depressed mood occurs suddenly during the day, determining “mood lability” (Starkman et al., 1981). Moreover, in patients with CS, depressed mood has been reported to be often characterized by hypersensitivity and oversentimentality, increased feeling of crying, short spells of sadness, and less frequently constant hopelessness, social withdrawal with feelings of discomfort in large groups, intermittent inability to experience pleasure, which rarely reaches the levels of persistent anhedonia, and less often self-accusatory or irrational guilt (Starkman et al., 1981; Starkman, 2013). Conversely, a minority of patients, particularly in the first phase of active hypercortisolism, experiences elevated mood with episodes of hyperactivity, increased ambition and restlessness that generally disappears with the time over disease progression (Starkman et al., 1981; Starkman, 2013). Suicidal thoughts (17%) and suicide attempts (5%) have also been described (Starkman et al., 1981; Starkman, 2013) and frequently minimized by patients (Haskett, 1985; Sonino and Fava, 2001). In patients with active hypercortisolism symptoms and signs involving the vegetative functions are frequently reported. Indeed, fatigue has been described in almost all patients and a decreased libido in about two-thirds (Starkman et al., 1981). Disturbance of appetite and sleep have also been described; particularly, an increased or decreased appetite have been found in about 34% and 20% of patients, respectively (Starkman et al., 1981) and sleep disturbances such as middle insomnia (patients awakened at least one during the night) in 69% of CS patients, late insomnia (patients awakened earlier than desired in the morning) in 57% and early insomnia (inability to fall asleep at bedtime) in 29% (Starkman et al., 1981; Starkman, 2013). In about one third of patients, it has also been reported an alteration in the frequency and type of dreams which became more bizarre and vivid (Starkman et al., 1981; Starkman, 2013). Within the clinical frame of depressive syndrome, patients may also experience cognitive impairments that will be detailed below.

**Table 1:** **Prevalence of major depression in patients with active CS (adapted from Sonino and Fava, 2001)**.

**References**	**Diagnosis**	**No. of PTS**	**Major depression: No. of PTS (%)**
Sonino et al., 1998	CD	162	88 (54)
Haskett, 1985	All forms	30	24 (80)
Hudson et al., 1987	CD	16	9 (56)
Loosen et al., 1992	CD	20	13 (65)
Sonino et al., 1993	CS excluding CD	20	10 (50)
Kelly, 1996	All forms	209	120 (57)
Dorn et al., 1995	All forms	33	17 (51)
Total		490	281 (57)

*CS, Cushing's syndrome; CD, Cushing's disease*.

A significant percentage (66%) of CS patients reported generalized anxiety or panic disorders more frequently described in the chronic and advanced stage of active hypercortisolism (Starkman et al., 1981; Starkman, 2013). Also bipolar disorders including maniac and hypomaniac episodes have been observed in about 30% of CS patients and may represent an early manifestation of CS (Haskett, 1985; Hudson et al., 1987).

Neurocognitive impairment has been reported in about two thirds of CS patients with variable degrees from mild to severe (Whelan et al., 1980). An impairment of memory has been reported in 83% of CS patients, consisting of difficulty in processing new information and forgetfulness of information such as appointments, names of people, location of objects, important dates in their personal, and/or medical histories (Starkman et al., 1981; Starkman, 2013). Impaired concentration has been reported in 66% of CS patients that particularly complain of mind-wandering when reading, watching television and during the course of conversations, a decreased ability to “focus their minds” and more in general inattention, distractibility, shortened attention span, difficulties with reasoning ability, and comprehension (Starkman et al., 1981; Starkman, 2013). Moreover, disturbances in “thinking” may occur. Indeed, patients with CS can experience episodes of rapid and scattered thinking, or slow thinking with blocks, that are described as a mind, which suddenly “becomes blank” (Starkman, 2013). In addition, impairment in verbal control, non-verbal, visual, and spatial abilities has also been reported in CS patients (Starkman et al., 1981; Sonino and Fava, 2001; Starkman, 2013).

## Neuropsychiatric disorders after disease remission

Several studies have reported that, after disease remission, the resolution of hypercortisolism, namely the normalization of cortisol secretion, is not constantly followed by the complete recovery of many clinical complications developed during the active disease; in particular, metabolic syndrome and the consequent cardiovascular risk have been found to partially persist after disease remission (Colao et al., 1999; Faggiano et al., 2003; Pivonello et al., 2005, 2007). Skeletal diseases, mainly osteoporosis, improve after disease remission, but the normalization of bone mass may require a long period of time or the use of specific treatments (Faggiano et al., 2001; Pivonello et al., 2007). Similarly, psychiatric and neurocognitive disorders generally improve after disease remission, but several studies reported that these disorders can persist, even long-term after the resolution of hypercortisolism and occasionally they can even exacerbate with the decrease and resolution of hypercortisolism. Consequently, this slow and partial recovery of different clinical alterations occurred during the active phase of the disease can contribute to the persistent impairment of QoL registered in CS patients after disease remission or cure (Sonino and Fava, 2001; Pivonello et al., 2007; Webb et al., 2008; Colao et al., 2012).

To date, the issue of whether remission of CS may completely resolve psychiatric and neurocognitive disorders remains controversial. Several studies demonstrated a significant improvement of psychiatric and neurocognitive disorders after resolution of hypercortisolism (Jeffcoate et al., 1979; Cohen, 1980; Kramlinger et al., 1985; Starkman et al., 1986; van der Lely et al., 1991; Verhelst et al., 1991; Kelly et al., 1996; Wolkowitz and Reus, 1999; Hirsch et al., 2000). In particular, Starkman and co-workers reported a significant improvement in depression scores of patients treated for CD, which were significantly correlated with the decrease in urinary cortisol excretion (Starkman et al., 1986). Different studies described significant improvements or even complete remission of depression, anxiety, irritability, psychosis, and neurocognitive impairment, after normalization of cortisol levels in CS patients treated with radiotherapy, surgery, or medical treatment (Jeffcoate et al., 1979; Cohen, 1980; Kramlinger et al., 1985; Starkman et al., 1986; van der Lely et al., 1991; Verhelst et al., 1991; Kelly et al., 1996; Dorn et al., 1997; Wolkowitz and Reus, 1999; Hirsch et al., 2000). Particularly, some studies have reported the effects of successful reduction of GCs excess on psychopathology. These studies demonstrate that both reduction of GCs synthesis with ketoconazole or metyrapone and blockade of the GC receptor with mifepristone positively affect psychopathology. Interestingly, mifepristione has been demonstrated to have therapeutic effects in patients with psychotic depression. Additionally, this drug can mitigate the weight gain associated with the use of antipsychotic drugs and improve cognitive dysfunction in bipolar depression (Howland, 2013). However, as mentioned above, more recently, several studies showed that neuropsychiatric improvement, after a successful treatment of CS, might be delayed and incomplete (Dorn and Cerrone, 2000; Sonino and Fava, 2001; Forget et al., 2002; Heald et al., 2004; Merke et al., 2005; Maheu et al., 2008; Tiemensma et al., 2010; Ragnarsson et al., 2012; Resmini et al., 2012; Ragnarsson and Johannsson, 2013). Indeed, both psychiatric and neurocognitive alterations may require long time to recover after remission of hypercortisolism and they may sometimes persist even after CS cure. Long-lasting impairments were reported in many domains of cognitive function including attention, visuospatial orienting, alerting, reasoning, working and speed memory, verbal fluency, reading, and executive functions (Forget et al., 2002; Tiemensma et al., 2010; Ragnarsson et al., 2012; Resmini et al., 2012). Indeed, the brain volume loss, which is considered the main responsible for the neurocognitive decline of active CS patients, has been demonstrated to be partially persistent after remission (Bourdeau et al., 2002), but further studies are still required to determine whether these alterations can fully revert after long-term remission or better after a time long enough to consider the patients definitively cured. Additionally, in patients long-term remitted from CS, proton magnetic resonance spectroscopy, measuring brain metabolites in the hippocampus, suggested neuronal dysfunction or loss and consequently a repair mechanism of glial proliferation (Resmini et al., 2013). These alterations indicated the persistence of a neuronal damage associated with negative effects on hippocampal volume and memory (Resmini et al., 2013). To investigate whether neuropsychiatric disorders may persist in all patients treated for pituitary tumors, regardless the type of hormone secretion, Heald and co-workers compared patients treated for CD with patients treated for acromegaly, macroprolactinomas, or non-functioning pituitary tumors. In this study patients treated for CD appeared to perceive themselves as being more depressed, fatigued, anxious, having poorer physical health and environmental, and social adjustment, showing significantly impaired psychological well-being and psychosocial functioning compared with all other pituitary tumors patients (Heald et al., 2004). All these observations seem to validate that CS, even after resolution of hypercortisolism, has long-lasting adverse effects on CNS, suggesting that depression, anxiety, and neurocognitive impairment, may still dominate the clinical picture even after disease remission. Interestingly, Dorn and co-workers firstly reported that in CS patients some psychiatric symptoms might also exacerbate with cortisol decrease (Dorn et al., 1997). Particularly, it has been reported that the frequency of panic attacks and suicidal ideation increased after CS remission in a subgroup of patients. This phenomenon has been suggested to be due to the relative GC deficiency, which seems to allow unrestrained increase in catecholamines (Dorn et al., 1997). Moreover, in children and adolescents with CS, Merke and co-workers reported a significant decline of cognitive function 1 year after surgical correction of hypercortisolism, despite a rapid resolution of cerebral atrophy was observed (Merke et al., 2005).

Overall these data highlight the importance of a long-term follow-up and careful periodic investigation of neuropsychiatric symptoms in patients with CS also after hypercortisolism resolution, and the importance to consider the management of these disorders as one of the essential outcomes of CS patients.

## Effects of neuropsychiatric disorders on quality of life

Considering the systemic and neuropsychiatric complications associated with hypercortisolism it is expected that CS patients have impairment in QoL (Colao et al., 2012). Indeed, impairment in QoL has been demonstrated both by generic and disease-specific questionnaires, namely Cushing QoL, which has been reported to be the most appropriate to measure QoL in patients with CS (Webb et al., 2008). Multiple physical and psychological factors can interfere with QoL in CS patients, but the results of the ERCUSYN study, a recent published web-based, multicentre, observational study from 23 European countries, showed that only depression resulted an independent predictor of a lower CS QoL score, suggesting that psychiatric disorders, and mainly depression, may play a pivotal role in affecting QoL in patients with CS (Valassi et al., 2011). Indeed, patients suffering from CS most often complain of changes in physical appearance, depression, emotional instability, fatigue and/or weakness, sleeping difficulties, neurocognitive problems, interference with family life, relations with their partner, and with school/work performance (Gotch, 1994; Colao et al., 2012). As main psychiatric and neurocognitive disorders, the impairment in QoL of patients with CS is only partially resolved after treatment of hypercortisolism and a longer-term follow-up showed that a residual impairment of QoL may persist after long-term disease remission (Lindsay et al., 2006; Wagenmakers et al., 2012). Indeed, patients in remission upon proper treatment for CS displayed significantly higher scores in depression (feeling tired, guilty, hopeless, unworthy, poor appetite, loss of interest), anxiety (being nervous, tense, panicky, difficulties falling asleep, or early morning awakening), and psychotic symptoms (feelings of persecution, delusions, hallucinations), with a generalized compromised QoL compared with healthy subjects (Sonino et al., 2006). Particularly in patients with CD, despite long-term remission, QoL appeared reduced compared with reference values from the literature, especially in the presence of pituitary insufficiencies (van Aken et al., 2005). Interestingly, also in children and adolescents, CS is associated with impaired QoL, with residual impairment 1-year after treatment, suggesting that QoL is another important parameter of outcome also in these patients and that the identification of factors that contribute to impair QoL may help to diminish the physical and psychological burden of disease in this population of patients (Keil et al., 2009).

## Conclusions

In conclusion, CS is frequently associated with an high prevalence of psychiatric and neurocognitive disorders and with a significant impairment in QoL. Major depression represents the most important and life-threatening psychiatric complication, occurring in approximately 50–60% of patients, but mania and anxiety are also reported in many cases. Additionally, neurocognitive disorders may occur both as a manifestation within the depressive syndrome clinical frame, and separately as isolated neurological disorder. It should be highlighted that psychiatric and mainly neurocognitive disorders, even long-term after CS remission, although generally improve, may persist, or even worsen, contributing to the persistent impairment of QoL registered also in patients treated for CS. Therefore, long-term follow-up and careful periodical investigation of psychiatric and neurocognitive symptoms should be always considered in the management of CS both in the active phase and after disease remission.

### Conflict of interest statement

The authors declare that the research was conducted in the absence of any commercial or financial relationships that could be construed as a potential conflict of interest.
